# Sex-specific chromatin states in mammalian fetal germ cells

**DOI:** 10.1186/1756-8935-6-S1-P45

**Published:** 2013-03-18

**Authors:** Bluma J Lesch, David C Page

**Affiliations:** 1 HHMI, Whitehead Institute, and Department of Biology, MIT, Cambridge, Massachusetts, 02142 USA

## Background

Male and female mammalian germ cells follow identical developmental trajectories for the first half of embryogenesis, during which time they also maintain a pluripotent-like state. Beginning around day 13.5 of embryogenesis (E13.5), male and female germ cells initiate dramatically different developmental programs: female germ cells enter meiotic prophase, while male germ cells enter a G_0_-like cell cycle arrest until after birth [1]. At this time, both male and female germ cells also lose the ability to establish pluripotent cell lines in culture [2]. As late as E12.5, male and female germ cells are morphologically identical, and few transcriptional differences can be detected [1,3]. To evaluate the coordination of sex-specific transcriptional states during this important interval in germ cell differentiation, we examined placement of the activating histone modification H3K4me3 and the repressive histone modification H3K27me3 in XX and XY murine germ cells before and during the initiation of sex differentiation.

## Materials and methods

Germ cells expressing an Oct4-EGFP transgene [4] were isolated by flow cytometry from male and female mouse embryos at 12.5 or 13.5 days post coitus. Cells from multiple embryos were pooled and split into equal parts for ChIP against H3K4me3 or H3K27me3, followed by high-throughput sequencing. ChIP-seq data was compared to preexisting RNA-seq data from E12.5 or E14.5 male and female gonads.

## Results

At day E12.5, at least 80% of H3K4me3 peaks associated with gene promoters were shared between male and female germ cells. Many genes marked by H3K4me3 in one sex but not the other at E12.5 were expressed specifically in germ cells of that sex one to two days later. Compared to H3K4me3, we found significantly more differences in placement of the H3K27me3 histone modification between males and females. Differences in H3K27me3 placement between sexes at E12.5 also corresponded to known differences in gene expression at later time points.

## Conclusions

Mammalian germ cells display sex-specific chromatin configurations prior to the appearance of significant sex differences in morphology and gene expression. These differences in chromatin state anticipate sex differences in gene expression at later time points.
